# Significance of serum antibodies against HPV E7, Hsp27, Hsp20 and Hp91 in Iranian HPV-exposed women

**DOI:** 10.1186/s12879-019-3780-2

**Published:** 2019-02-12

**Authors:** Amitis Ramezani, Arezoo Aghakhani, Sepehr Soleymani, Anahita Bavand, Azam Bolhassani

**Affiliations:** 1 Clinical Research Department, Pasteur Institute of Iran, Tehran, Iran; 2 Department of Hepatitis and AIDS, Pasteur Institute of Iran, Tehran, Iran

**Keywords:** HPV, E7, Serological marker, Heat shock protein, Hp91

## Abstract

**Background:**

Among different types of human papillomavirus (HPV), types 16 and 18 were known to be high-risk agents causing mainly cervical cancer. Up to now, the potential of HPV E7 protein has been proved as a diagnostic marker of cervical cancer. Moreover, the levels of anti-heat shock protein (Hsp) and anti-high mobility group box-1 (HMGB1) antibodies in cancer patients have been useful in tumor diagnosis. The goal of the present study was to determine the efficiency of the potential serologic markers including HPV E7, Hsp20, Hsp27 proteins and Hp91 peptide in Iranian HPV-exposed women, for the first time.

**Methods:**

At first, the recombinant HPV E7, Hsp20 and Hsp27 proteins were expressed in *E. coli* system, and purified by affinity chromatography under native conditions. Then, antibody responses were detected against the recombinant proteins as well as Hp91 peptide as potential markers in 49 Iranian women who were seropositive for HPV-16 and 18 L1 capsids (i.e.*,* HPV-exposed women) and 49 controls using indirect ELISA.

**Results:**

Our data indicated that the seroreactivities of women exposed to HPV16, HPV18 and both of them against the recombinant E7, Hsp20, Hsp27 proteins and Hp91 peptide were significantly higher than those in control group (*p* < 0.05 for HPV16 or HPV18; *p* < 0.01 for both of them versus all markers). HPV-exposed women with high antibody responses to HPV-16 and 18 L1 capsids as a commercial biomarker had significant seroreactivity to HPV-16 and 18 E7 and Hsp27 (*p* < 0.05). The recombinant E7 and Hsp27 proteins showed higher efficiency than Hsp20 and Hp91 for detection of individuals exposed to HPV infections (*p* < 0.05).

**Conclusion:**

Generally, the levels of serum E7 and Hsp27 were increased in HPV-16 and 18 L1- seropositive women suggesting their potential value as a diagnostic marker for HPV infections.

**Electronic supplementary material:**

The online version of this article (10.1186/s12879-019-3780-2) contains supplementary material, which is available to authorized users.

## Study significance

The studies indicated that most high-risk HPV infections in young subjects are transient and have little long-term significance. Only a small proportion of infected individuals (~ 5–10%) fail to clear HPV and suffer from persistent infections. Thus, serological assays are important for detecting anti-HPV antibodies and determining the subjects with previous exposure to HPV, who may be protected against subsequent HPV infection. In the current study, we showed that HPV E7 and Hsp27 can be considered as promising biomarkers similar to HPV L1 for determination of HPV-16 and 18-exposed women.

## Background

Human papillomavirus (HPV) infection has been recognized as the main etiologic factor in the development of different cancers [1]. The HPV types have been currently determined up to 225 types [2]. Among the high-risk HPVs, types 16 and 18 are responsible for the majority of cervical cancer in the world especially in Iran [1, 3]. The persistent HPV infections generate E6 and E7 oncoproteins which promote cell proliferation and carcinogenesis leading to increased levels of host antibodies [4]. As known, HPV DNA test along with routine Pap smear could reduce colposcopy rate in ASCUS patients [5]. A report recommended an organized cervical screening with HPV DNA testing for women in Iran, beginning at age 35 and repeated every 10 or 5 years [6]. However, biomarkers are required to select patients for colposcopy screening in low and middle income countries where DNA and cytology testing are too expensive. Moreover, the assessment of antibody levels against HPV antigens has been important to determine the natural history of infection and efficiency of vaccination [7]. The studies indicated that a large proportion of high-risk HPV infections in young women are spontaneously removed and have short-term effects. Indeed, low rates of infected women, approximately 5–10%, suffer from persistent HPV infections. Therefore, detection of anti-HPV antibodies is important for determination of the overall HPV exposure rate in a population [8–12]. There are various serologic markers for HPV infections such as detection of HPV DNA in tumors, antibody assay against HPV L1, E6 and E7 proteins, and the levels of p16 protein [13–16]. The antibodies against HPV L1 were considered as markers of cumulative exposure to the virus [17]. Indeed, the antibody response in host to HPV L1 is poor and may remain for years as an indicator for past infection but not malignancy. Some studies showed that the presence of E7-specific antibodies was associated with an increased relative risk for cervical cancer that may be detected up to 5 years prior to diagnosis [7]. Recently, combination of HPV markers is preferred to predict survival among individuals with HPV infections [18].

As known, heat shock proteins (HSPs) are present in all prokaryotic and eukaryotic organisms. These proteins are divided into various groups based on their molecular weight such as Hsp60, Hsp70, Hsp90, Hsp110 and small Hsps (e.g.*,* Hsp27 and Hsp20) [19]. Heat shock proteins are overexpressed in a wide range of human cancers and involved in recognition by the immune system [20, 21]. Among heat shock proteins, small HSPs are highly conserved proteins among all species which have a conservative α-crystallin domain (~ 90 amino acid residues). Some small heat shock proteins are expressed in all human tissues including HspB1 (Hsp27), αB-crystallin (HspB5), HspB6 (Hsp20) and HspB8 [22, 23]. For example, small Hsp27 is a multifunctional protein which acts as a protein chaperone and an antioxidant and plays a role in the inhibition of apoptosis. Hsp27 is a biomarker of infections and also a therapeutic target in cancer [24]. A study showed that the host Hsps such as Hsp25, Hsp60, Hsp70 and Hsp90 were used as potential biomarkers for the diagnosis of tuberculous meningitis (TBM) [25]. Another study indicated that the serum level of Hsp27 as a potential marker was increased in Egyptians with Type 2 Diabetes [26]. These reports showed that the levels of Hsps can change in infectious and non-infectious diseases.

On the other hand, the high-mobility group box-1 (HMGB1) protein known as amphoterin, is a highly conserved, non-histone nuclear protein expressed in almost all eukaryotic cells. Recent clinical studies have shown that HMGB1 is a potential diagnostic or prognostic biomarker in a variety of inflammatory disorders and cancers [27, 28]. The cytokine-inducing part of the HMGB1 molecule is among the first 20 amino acids of the B-box domain (aa 89–108) known as Hp91 peptide [29, 30]. The studies indicated that the Hp91 peptide is a potent inducer for generation of Th1-type immune responses [31, 32].

In this study, at first, the recombinant (r) HPV E7, Hsp20 and Hsp27 proteins were expressed in *Escherichia coli* (*E. coli*) as a His-tag protein and purified using affinity chromatography. *E. coli* is the most popular expression system for production of the recombinant proteins. This system has some advantages compared to other systems such as low cost, high yield, easy purification, a large number of expression plasmids and strains, and many cultivation strategies [33].

After production of the recombinant proteins, the seroreactivities of Iranian women who were seropositive for HPV-16 and 18 L1 capsids as mono- and co-infection (as previously reported; 26) were evaluated against the rE7, rHsp20, rHsp27 proteins as well as Hp91 peptide as diagnostic markers. Indeed, we compared the efficiency of HPV-16 and 18 L1 capsids with HPV-16 and 18 E7, Hsp27, Hsp20 and Hp91 for detection of anti-HPV IgG antibodies using indirect ELISA in Iranian women exposed to HPV infections not diagnosed with cervical cancer.

Briefly, our goal was to determine novel and effective markers instead of L1 capsid-based commercial kit for diagnosis of HPV-exposed subjects. We used the samples recognized by L1 kits whether a specific HPV marker (i.e.*,* E7) along with a non-specific marker (i.e.*,* Hsp27, Hsp20 or Hp91) possess high efficiency for diagnosis of HPV-exposed women. On the other hand, this study confirms whether Iranian women who were seropositive for HPV-16 and 18 L1 capsids are really HPV-infected women at present. However, it will be required to design new kits with suitable efficacy and low cost for serological tests.

## Methods

### Study population

Serum samples were collected from 49 Iranian women (with no previous treatment, vaccination and immunodeficiency disorders) who were seropositive for HPV-16 (22 cases, group 1), HPV-18 (12 cases, group 2) and both HPV-16 and 18 (15 cases, group 3) L1 capsids as observed in our previous study (Additional file 1, Ref. [34]) and 49 control women. All the subjects were recruited from outpatients attending to hospitals in Tehran, Iran, for routine checkup or mild diseases such as common cold; 34). These samples were examined to detect antibodies against the HPV-16 and 18 E7, Hsp20 and Hsp27 proteins, and Hp91 peptide. Also, 49 Iranian HPV-16 and 18 L1 seronegative cases (healthy individuals without HPV infection, other viral infections, e.g.*,* HIV, HCV, HBV, HGV, HSV, bacterial infections, e.g.*,* TB and non-infectious disorders, e.g.*,* cancers) and one commercial standard sera (USA origin: Innovative Research, INC, without any infection, a validated control) were enrolled as control group for evaluation of anti-HPV antibody in sera against the E7, Hsp20, Hsp27 proteins and Hp91 peptide (Pateint diagram in Additional file 1). In this study, the mean age of Iranian patients was 27.44 yr. (range, 11 to 35 yr), and the mean age of Iranian controls was 30.20 yr. (range, 23 to 35 yr). All participants gave written informed consent and the Pasteur Institute of Iran Ethics Committees cleared and approved the study protocol.

### Expression and purification of the recombinant HPV16 E7 protein

For generation of the recombinant HPV16 E7 protein (rE7), the *E. coli* M15 strain harboring E7 gene (pQE-E7, [16]) was grown to an optical density of 0.7 to 0.8 at 600 nm and protein expression was induced by adding 1 mM IPTG at 37 °C. Protein samples were analyzed by SDS-PAGE at 4 h after induction. Then, the soluble fraction of rE7 was purified by affinity chromatography on Ni-NTA resin (Qiagen protocol) under native conditions using imidazole buffer. The purified E7 protein fractions were identified by Western Blot (WB) analysis using anti-His tag antibody (Abcam), dialyzed against PBS1X, and detected by LAL assay (QCL-1000, Lonza) for the LPS contamination. This contamination was lower than 0.2 EU/mg which was suitable for the next use in ELISA. Moreover, for Western Blotting, the immunoreactive protein bands were visualized on nitrocellulose membrane (Millipore, USA) using peroxidase substrate 3, 3′-diaminobenzidine (DAB, Sigma). Finally, the protein concentration was assessed by NanoDrop Spectrophotometer and stored at − 70 °C. The HPV-18 E7 protein was available in laboratory as previously provided by our group.

### Cloning, expression and purification of the recombinant Hsp27 protein

At first, the full length of human Hsp27 gene (Accession No: NM_001540) was synthesized in a prokaryotic expression vector (pQE30-Hsp27, Biomatik Co., Canada). To generate pET-Hsp27, the Hsp27 fragment was subcloned from pQE30-Hsp27 into the pET-23a in *Nhe*I*/ Sal*I cloning site. Next, the *E. coli* Rosetta strains were transformed with the recombinant pET-Hsp27 plasmid and the clones were selected on LB-agar plate. These bacterial clones were grown to an optical density of 0.6–0.7 at 600 nm in Ty2x medium and protein *expression* was i*nduced by 1 mM* IPTG. The cell pellets were harvested at 4 h after induction and analyzed by 12% SDS-PAGE. The recombinant Hsp27 protein was purified by affinity chromatography under native conditions using imidazole buffer according to the manufacturer’s instructions (Qiagen), and identified by Western Blot analysis using anti-His tag antibody. Then, the purified protein was dialyzed against PBS1X and detected by LAL assay for the LPS contamination. This contamination was lower than 0.5 EU/mg which was suitable for the next use in ELISA. Finally, the protein concentration was assessed by NanoDrop Spectrophotometer and stored at − 70 °C.

### Cloning, expression and purification of the recombinant Hsp20 protein

The full length of human Hsp20 gene (Accession No: NM_144617) was synthesized in a prokaryotic expression vector (pQE30-Hsp20, Biomatik Co., Canada). To generate pET-Hsp20, the Hsp20 fragment was subcloned from pQE30-Hsp27 into the pET-23a in *Nhe*I*/ Hind*III cloning site. All expression and purification protocols were performed similar to the production of Hsp27 as mentioned above. Herein, the endotoxin contamination was less than 0.4 EU/mg for Hsp20 *protein* as detected by *LAL assay* which was suitable for the next use in ELISA.

### Preparation of Hp91 peptide.

The Hp91 peptide (DPNAPKRPPSAFFLFCSE) with an N-terminal biotin was synthesized and purified by BioMatik Co. (Canada; [32]). The peptides were dissolved in PBS 1X for ELISA experiments.

### Detection of IgG antibodies against rE7, rHsp27, rHsp20 proteins and Hp91 peptide

For detection of IgG antibodies against the recombinant proteins, 96-microwell plates (Greiner) were coated overnight at 4 °C with 100 μl of the recombinant HPV E7 (rE7, 5 μg/ml), Hsp27 (rHsp27, 5 μg/ml), Hsp20 (rHsp20, 5 μg/ml) proteins as well as Hp91 peptide (10 μg/ml). Then, the plates were blocked with 1% BSA in PBS for 2 h at 37 °C, and incubated with 100 μl of human sera diluted 1:100 in blocking buffer containing 0.05% (*v*/v) Tween 20 for 2 h at 37 °C. The bound human antibodies were detected by goat anti-human IgG conjugated to horseradish peroxidase (HRP, Sigma, 1:10000) and then addition of 3,3′,5,5’-Tetramethylbenzidine (TMB, Sigma) as the substrate. All washing steps were done with PBS1X containing 0.05% Tween 20. The enzyme reaction was stopped by adding 50 μl of 1 M sulphuric acid and the absorbance was determined at 450 nm.

### Statistical analysis

Statistical analysis was performed using Prism 5.0 software (GraphPad). One-way ANOVA was used to analyze the differences in the levels of antibody production. For all analyses, *p* < 0.05 was considered statistically significant.

## Results

### Study population

Iranian subjects who were seropositive for HPV-16 and 18 L1 capsids were divided into three groups: seropositive for HPV-16 (group 1, 22 cases), seropositive for HPV-18 (group 2, 12 cases), and seropositive for both HPV-16 and HPV-18 infections (group 3, 15 cases). Indeed, seroprevalence was 45% for HPV16 L1, 24% for HPV18 L1 and 31% for both HPV16 and HPV18 L1 in 49 Iranian subjects.

### Generation of the recombinant HPV16E7, Hsp20 and Hsp27 proteins

**Fig. 1:**
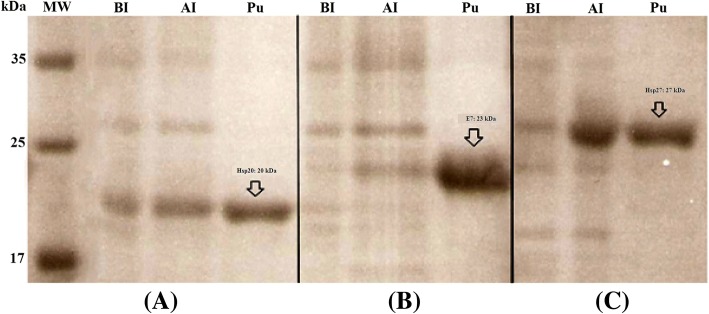
Expression and purification of the recombinant proteins: The recombinant human Hsp20 (**a**), E7 (**b**), and human Hsp27 (**c**) proteins were run on a polyacrylamide gel electrophoresis and stained by coomassie blue. MW is the molecular weight marker (prestained protein ladder: 10–170 kDa, Fermentase). Samples of before induction (BI), after induction (AI), and the purified recombinant proteins (Pu) were shown in figure for each protein, respectively

The recombinant HPV E7, human Hsp20 and human Hsp27 proteins were successfully expressed in *E. coli* strains. The rE7, rHsp20 and rHsp27 proteins migrated as the 23, 20 and 27 kDa bands in SDS-PAGE, respectively. Their purification was performed under native conditions using affinity chromatography (Fig. 1). The purified proteins were identified by Western Blotting as shown in Additional file 2. The NanoDrop Spectrophotometer showed that the recombinant proteins had a concentration range between 0.8 and 1 mg/ml.

### Detection of HPV E7, Hsp27, Hsp20, Hp91-specific antibodies by ELISA

**Fig. 2:**
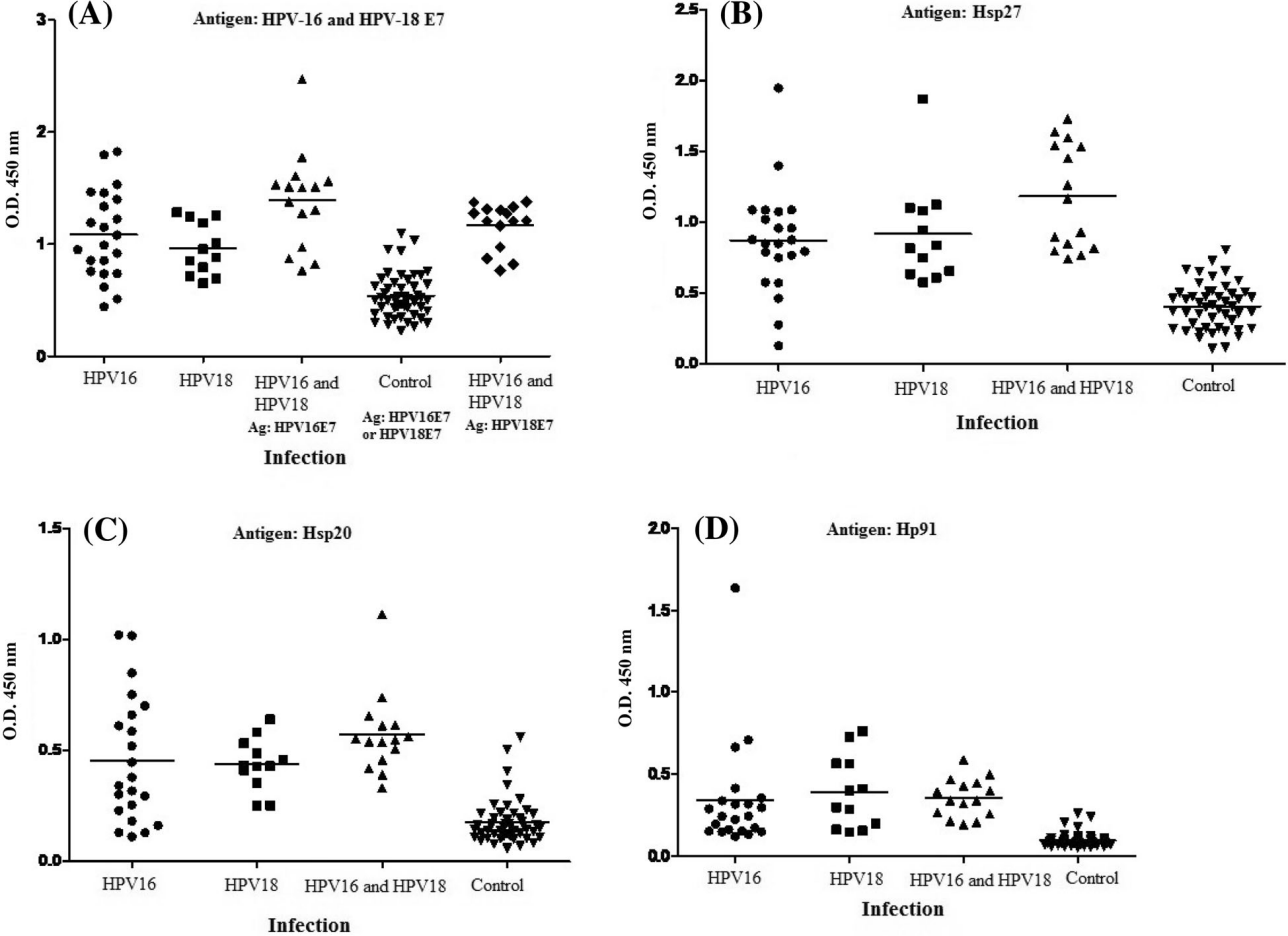
Analysis of IgG antibody levels with respect to rE7 (**a**), rHsp27 (**b**), rHsp20 (**c**), and rHp91 (**d**) coating antigens in HPV-exposed women compared to control group using ELISA. The horizontal line represents the mean value of optical density in respect to each antigen

**Fig. 3:**
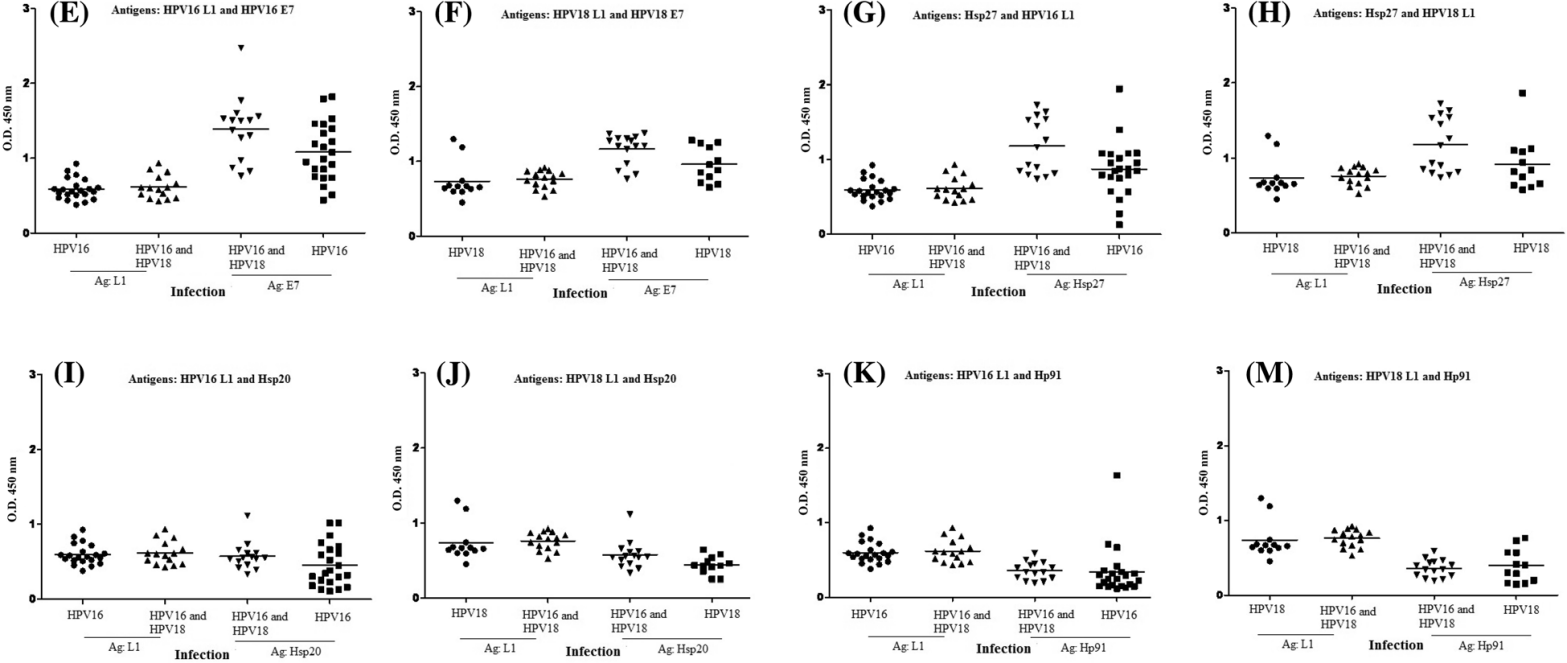
Analysis of IgG antibody levels against rE7 (E&F), rHsp27 (G&H), rHsp20 (I&J), and rHp91 (K&M) coating antigens as compared to HPV-16 and 18 L1 antigens in HPV-exposed women using ELISA. The horizontal line represents the mean value of optical density in respect to each antigen

The frequency of antibodies to HPV E7, Hsp20, Hsp27 proteins and Hp91 peptide was determined in 49 serum samples from Iranian women who were seropositive for HPV-16 and 18 L1 capsids [34] as well as controls. The mean absorbance values for rE7, rHsp20, rHsp27 proteins and Hp91 peptide in HPV-16-exposed women were 1.083 ± 0.390, 0.455 ± 0.284, 0.873 ± 0.374 and 0.338 ± 0.329, respectively. The levels of antibodies against all recombinant proteins in HPV-exposed women were significantly higher than those in control group (*p* < 0.001). The results showed that the levels of antibodies against rE7, rHsp20, rHsp27 proteins and Hp91 peptide in HPV-16 exposed women did not show any statistically differences with HPV-18 exposed women (*p* > 0.05). In addition, the levels of antibodies against rHsp20 and Hp91 peptide in HPV-18-exposed women showed no statistically differences with women exposed to both HPV-16 and HPV-18 (*p* > 0.05). In contrast, the levels of antibodies against the rE7 and rHsp27 in women exposed to both HPV-16 and HPV-18 were higher than women exposed to HPV-16 or HPV-18 (*p* < 0.05). Figure 2 shows the differences between the seroreactivities in HPV-exposed individuals and controls against rE7, rHsp20, rHsp27 proteins and Hp91 peptide. To determine the serum reactivity for each recombinant protein, and peptide, a cut-off value was calculated by considering the mean absorbance values of control sera plus two standard deviations (mean ± 2SD). Overall, 50% of the serum samples from women with seroposivity to HPV-16 L1 antibody reacted in four assays as anti-E7, anti-Hsp20, anti-Hsp27, and anti-Hp91 antibody positive and 55% of the HPV-18 sera recognizing all recombinant proteins. Also, 75% of the serum samples from women with seroposivity to HPV-16 and 18 L1 antibodies were positive to all recombinant proteins. Indeed, HPV E7 and Hsp27 were more effective biomarkers than Hsp20 and Hp91 for detection of both HPV infections. On the other hand, the efficiency of HPV-16 and 18 L1 capsids was compared with HPV-16 and 18 E7, Hsp20 and Hsp27 proteins as well as Hp91 peptide for determination of women exposed to HPV16, HPV18 and both infections. The results indicated that the HPV E7 and Hsp27 can be considered as promising biomarkers similar to HPV L1 for determination of HPV-16 and 18-exposed women (Fig. 3).

## Discussion

In the current study, the presence of human antibodies to HPV E7 protein, small heat shock proteins 20 and 27, and Hp91 peptide was indicated by ELISA in HPV-exposed Iranian women. The recombinant proteins expressed in *E. coli* were used as antigens in ELISA for screening 49 serum samples obtained from individuals who were seropositive for HPV-16 (22 cases), HPV-18 (12 cases) and both HPV-16 and HPV-18 (15 cases) L1 capsids as well as control group (without any infections, 34). SDS-PAGE analysis of IPTG-induced cell lysates showed the presence of a prominent protein band of 23, 20 and 27 kDa for E7, Hsp20 and Hsp27, respectively that was not detectable in non-induced cell lysates. Then, the small heat shock proteins and Hp91 peptide besides viral oncogenic protein (E7) could be used to screen the seropositive individuals for HPV L1 capsids. Our ELISA findings indicated that HPV-exposed women with high antibody response to HPV-16 and 18 L1 had significant seroreactivity to HPV-16 and 18 E7 and Hsp27.

Considering the importance of detecting HPV infection (i.e.*,* exposure rate to high-risk HPV types), a variety of methods was developed for these purposes [35]. The studies showed that the anti-HPV16 E7 antibody could be used as a blood biomarker for HPV infections [4]. Moreover, antibodies against HPV virus-like particles (VLPs) were considered as a marker of HPV infection, and were associated with HPV-related disease, but not as strongly as E6 and E7 antibodies [36]. A number of protein biomarkers are currently available to assist in screening HPV infections [37, 38]. The HPV antigens have been generated through the expression and purification of the recombinant proteins in different expression systems for vaccine development and/or detection of antibodies in human sera, saliva and cervical mucus using ELISA method [39]. The ELISA method is the main tool used for clinical diagnosis and screening large populations as well as evaluating the immunological responses to different HPV antigens. In the current study, we also generated the recombinant HPV16 E7, Hsp20 and Hsp27 proteins as an antigen under native procedures for the next use in ELISA.

As known, E6 and E7 transforming proteins are consistently expressed in HPV life cycle [39–41]. Several studies have shown the induction of antibody responses against E6 and E7 oncoproteins in patients with cervical cancer higher than in healthy subjects [39, 42, 43]. These studies and other reports usually indicated HPV seroreactivity for cancer patients in early or late stage; but our study showed a high seroreactivity in women-exposed to high-risk HPV types (i.e.*,* HPV L1-seropositive cases, 34) compared to un-infected women (i.e.*,* HPV L1-seronegative cases as controls) against the recombinant HPV E7 protein. In general, the detection of HPV-specific IgG in serum is a useful assay to determine previous and current HPV infection status [44].

On the other hand, heat shock proteins are highly conserved, but their expression levels between individuals were reported to differ widely for HSP60, HSP70 and HSP90 [45]. *The studies showed that HSP70 is a promising tumor marker for both diagnostic and therapeutic applications* [46, 47]*.* Moreover, several reports showed high levels of Hsp27 in sera of patients with different types of cancers by ELISA [20, 45, 48–53]. The studies showed that Hsp27 synthesis was enhanced during the early stages of endometrial cancer development. Antibodies to the Hsp27 were present in some women with ovarian and endometrial cancers but not in healthy women [53]. In addition, Hsp27 has been implicated in cardiovascular disease and atherosclerosis as a potential biomarker of disease and injury [24, 54, 55]. In this line, our study indicated the diagnostic capacities of Hsp27 and Hsp20 in women who were seropositive for HPV-16 and HPV-18 L1. We showed that the levels of Hsp27-specific antibody were significantly higher than the levels of Hsp20-specific antibody in individuals exposed to high-risk HPV types.

Recent clinical studies have shown that HMGB1 is a potential diagnostic or prognostic biomarker in a variety of inflammatory disorders and cancers. For instance, the serum level of HMGB1 was significantly elevated in benign and malignant asbestos-related diseases (ARDs) [28]. Other results demonstrated that HMGB1 levels were increased in patients with Crimean-congo hemorrhagic fever virus (CCHFV), Dobrava virus (DOBV) or Puumala virus (PUUV) infections. Indeed, HMGB1 could be considered as a potential biomarker for severe hemorrhagic fevers [56]. Furthermore, the serum HMGB1 level was increased by 1.5-fold in patients with colorectal carcinoma compared to those in healthy controls. Indeed, the diagnostic accuracy of HMGB1 for stage I cancer was significantly higher than that of carcinoembryonic antigen (CEA). The combination of HMGB1 and CEA led to increase significantly the overall diagnostic sensitivity compared to CEA alone [57, 58]. In this study, we used HMGB1-derived Hp91 peptide for detection of HPV infections in Iranian women who were seropositive for HPV L1 capsid. Our results showed that anti-Hp91 antibody was generated in HPV-exposed women significantly higher than HPV-uninfected women, but however, the responses were poor compared to E7, Hsp27 and L1-specific antibodies.

Generally, the use of commercial HPV L1 kit is efficient to determine HPV-exposed subjects, but there is a major limitation including cost and HPV type. This kit can effectively detect HPV types 16 and 18, thus the development of other markers will be necessary for this purpose especially in resource-poor countries. Our report demonstrated that oncological/viral biomarker (E7) associated with Hsp27 may be beneficial in the detection of individuals exposed to high-risk HPV types. It was interesting that individuals with strong anti-L1 antibody level showed high anti-E7 and anti-Hsp27 antibodies suggesting similar efficiency of these proteins as HPV biomarkers. Regarding the results, the combination of non-specific markers (i.e.*,* Hsps or Hp91) with specific markers (i.e.*,* HPV E7) could enhance significantly the overall diagnostic sensitivity as compared to each marker, alone. However, it is required to evaluate these markers in larger populations from different regions.

## Conclusions

In summary, women exposed to both HPV-16 and 18 infections showed higher antibody responses than women exposed to HPV-16 or HPV-18 against E7 and Hsp27, but not against L1, Hsp20 and Hp91. Moreover, no significant difference was observed in seroreactivities between HPV-16 and HPV-18-exposed women against Hsp20, Hsp27 and Hp91. Generally, the levels of serum E7 and Hsp27 were increased in women exposed to HPV-16, HPV-18 as mono- and co-infection suggesting their potential utility as a diagnostic marker for HPV infections. These markers can be used as good aims for resource-poor countries.

## Additional files

Additional file 1: Schematic representation of study population. (JPG 51 kb)

Additional file 2: Identification of the purified proteins by Western Blot analysis using anti-His antibody: lane 1: Hsp27, lane 2, Hsp20; lane 3, E7; MW is the molecular weight marker (prestained protein ladder: 10–170 kDa, Fermentase). (JPG 78 kb)

## Abbreviations

ARDs: Asbestos-related diseases
ASCUS: Atypical squamous cells of undetermined significance
CCHFV: Crimean-congo hemorrhagic fever virus
CEA: Carcinoembryonic antigen
DOBV: Dobrava virus
HMGB1: High mobility group box-1; r: recombinant
HPV: Human papillomavirus
HSP: Heat shock protein
PUUV: Puumala virus
VLP: Virus-like particles

## References

[1] Fontecha N, Basaras M, Hernáez S, Andía D, Cisterna R (2016). Assessment of human papillomavirus E6/E7 oncogene expression as cervical disease biomarker. BMC Cancer.

[2] Muhr LSA, Eklund C, Dillner J (2018). Towards quality and order in human papillomavirus research. Virology.

[3] Bruni L, Barrionuevo-Rosas L, Albero G, Aldea M, Serrano B, Mena M. Human papillomavirus and related diseases in the world. Summary Report 27 July 2017. ICO/IARC Information Centre on HPV and Cancer (HPV Information Centre). 2017; 1–325.

[4] Inan H, Wang S, Inci F, Baday M, Zangar R, Kesiraju S, Anderson KS, Cunningham BT, Demirci U (2017). Isolation, detection, and quantification of cancer biomarkers in HPV-associated malignancies. Sci Rep.

[5] Nalliah S, Karikalan B, Kademane K (2015). Multifaceted usage of HPV related tests and products in the management of cervical cancer: a review. Asian Pac J Cancer Prev.

[6] Nahvijou A, Daroudi R, Tahmasebi M, Amouzegar Hashemi F, Rezaei Hemami M, Akbari Sari A, Barati Marenani A, Zendehde K (2016). Cost-effectiveness of different cervical screening strategies in Islamic Republic of Iran: a middle-income country with a low incidence rate of cervical cancer. PLoS One.

[7] Ewaisha R, Panicker G, Maranian P, Unger ER, Anderson KS (2017). Serum immune profiling for early detection of cervical disease. Theranostics.

[8] Hildesheim A, Schiffman MH, Gravitt PE, Glass AG, Greer CE, Zhang T, Scott DR, Rush BB, Lawler P, Sherman ME (1994). Persistence of type specific human papillomavirus infection among cytologically normal women. J Infect Dis.

[9] Ho GY, Burk RD, Klein S, Kadish AS, Chang CJ, Palan P, Basu J, Tachezy R, Lewis R, Romney S (1995). Persistent genital human papillomavirus infection as a risk factor for persistent cervical dysplasia. J Natl Cancer Inst.

[10] Schulze MH, Völker FM, Lugert R, Cooper P, Hasenclever K, Groß U, Pfister H, Silling S (2016). High prevalence of human papillomaviruses in Ghanaian pregnant women. Med Microbiol Immunol.

[11] Schiffman MH, Bauer HM, Hoover RN, Glass AG, Cadell DM, Rush BB, Scott DR, Sherman ME, Kurman RJ, Wacholder S (1993). Epidemiologic evidence showing that human papillomavirus infection causes most cervical intraepithelial neoplasia. J Natl Cancer Inst.

[12] Schiffman M, Safaeian M, Wentzensen N (2009). The use of human papillomavirus seroepidemiology to inform vaccine policy. Sex Transm Dis.

[13] Yim EK, Park JS (2006). Biomarkers in cervical cancer. Biomark Insights.

[14] Chai RC, Lambie D, Verma M, Punyadeera C (2015). Current trends in the etiology and diagnosis of HPV-related head and neck cancers. Cancer Medicine.

[15] Tornesello ML, Buonaguro L, Giorgi-Rossi P, Buonaguro FM. Viral and cellular biomarkers in the diagnosis of cervical intraepithelial neoplasia and cancer. BioMed Res Intl. 2013;2013:1-10. 10.1155/2013/519619 PMC3872027 24383054

[16] Bolhassani A, Zahedifard F, Taslimi Y, Taghikhani M, Nahavandian B, Rafati S (2009). Antibody detection against HPV16E7 and Gp96 fragments as biomarkers in cervical cancer patients. Indian J Med Res.

[17] Combes JD, Pawlita M, Waterboer T, Hammouda D, Rajkumar T, Vanhems P, Snijders P, Herrero R, Franceschi S, Clifford G (2014). Antibodies against high-risk human papillomavirus proteins as markers for invasive cervical cancer. Int J Cancer.

[18] Liang C, Marsit CJ, McClean MD, Nelson HH, Christensen BC, Haddad RI, Clark JR, Wein RO, Grillone GA, Houseman EA, Halec G, Waterboer T, Pawlita M, Krane JF, Kelsey KT (2012). Biomarkers of HPV in head and neck squamous cell carcinoma. Cancer Res.

[19] Kuppusamy P, Govindan N, Yusoff MM, Ichwan SJA. Proteins are potent biomarkers to detect colon cancer progression. Saudi Journal of Biological Sciences. 2014:1–10. 10.1016/j.sjbs.2014.09.017 PMC5562385 28855814

[20] Ciocca DR, Calderwood SK (2005). Heat shock proteins in cancer: diagnostic, prognostic, predictive, and treatment implications. Cell Stress Chaperones.

[21] Bolhassani A, Rafati S (2008). Heat-shock proteins as powerful weapons in vaccine development. Expert Rev Vaccines.

[22] Basha E, O'Neill H, Vierling E (2012). Small heat shock proteins and alpha-crystallins: dynamic proteins with flexible functions. Trends Biochem Sci.

[23] Bakthisaran R, Tangirala R, Rao CM (2015). Small heat shock proteins: role in cellular functions and pathology. Biochim Biophys Acta.

[24] Vidyasagar A, Wilson NA, Djamali A (2012). Heat shock protein 27 (HSP27): biomarker of disease and therapeutic target. Fibrogenesis Tissue Repair.

[25] Shekhawat SD, Purohit HJ, Taori GM, Daginawala HF, Kashyap RS (2016). Evaluation of host Hsp(s) as potential biomarkers for the diagnosis of tuberculous meningitis. Clin Neurol Neurosurg.

[26] Mahgoub S, Youns M, Bassyouni A, Hassan Z (2012). Serum levels of heat shock protein 27 as a potential marker of diabetic nephropathy in Egyptians with type 2 diabetes. Journal of Applied Pharmaceutical Science.

[27] Eguchi A, Wree A, Feldstein AE (2014). Biomarkers of liver cell death. J Hepatol.

[28] Ying S, Jiang Z, He X, Yu M, Chen R, Chen J, Ru G, Chen Y, Chen W, Zhu L, Li T, Zhang Y, Guo X, Yin X, Zhang X, Lou J. Serum HMGB1 as a potential biomarker for patients with asbestos-related diseases. Hindawi Disease Markers. 2017:1–9. 10.1155/2017/5756102 PMC5350493 28348451

[29] Harris HE, Andersson U (2004). The nuclear protein HMGB1 as a pro-inflammatory mediator. Eur J Immunol.

[30] Gnanasekar M, Kalyanasundaram R, Zheng G, Chen A, Bosland MC, Kajdacsy-Balla A. HMGB1: a promising therapeutic target for prostate cancer. Hindawi Publishing Corporation, Prostate Cancer. 2013:1–8. 10.1155/2013/157103 PMC3666291 23766911

[31] Saenz R, Souza Cda S, Huang CT, Larsson M, Esener S, Messmer D (2010). HMGB1-derived peptide acts as adjuvant inducing immune responses to peptide and protein antigen. Vaccine.

[32] Saenz R, Futalan D, Leutenez L, Eekhout F, Fecteau JF, Sundelius S, Larsson M, Hayashi T, Minev B, Carson D, Esener S, Messmer B, Messmer D (2014). TLR4-dependent activation of dendritic cells by an HMGB1-derived peptide adjuvant. J Transl Med.

[33] Rosano GL, Ceccarelli EA (2014). Recombinant protein expression in *Escherichia coli*: advances and challenges. Front Microbiol.

[34] Aghakhani A, Mamishi S, Sabeti S, Bidari-Zerehpoosh F, Banifazl M, Bavand A, Ramezani A (2017). Gender and age-specific seroprevalence of human papillomavirus 16 and 18 in general population in Tehran. Iran Med Microbiol Immunol.

[35] Abreu ALP, Souza RP, Gimenes F, Consolaro MEL (2012). A review of methods for detect human papillomavirus infection. Virol J.

[36] Kreimer AR, Clifford GM, Snijders PJF, Castellsague X, Meijer CJLM, Pawlita M, Viscidi R, Herrero R, Franceschi S (2005). HPV16 semiquantitative viral load and serologic biomarkers in oral and oropharyngeal squamous cell carcinomas. Int J Cancer.

[37] Brown CA, Bogers J, Sahebali S, Depuydt CE, De Prins F, Malinowski DP (2012). Role of protein biomarkers in the detection of high-grade disease in cervical cancer screening programs. Hindawi Publishing Corporation, Journal of Oncology.

[38] Sahasrabuddhe VV, Luhn P, Wentzensen N (2011). Human papillomavirus and cervical cancer: biomarkers for improved prevention efforts. Future Microbiol.

[39] Gutiérrez-Xicoténcat L, Plett-Torres T, Madrid-González CL, Madrid-Marina V (2009). Molecular diagnosis of human papillomavirus in the development of cervical cancer. Salud Publica Mex.

[40] Muller M, Viscidi RP, Sun Y, Guerrero E, Hill PM, Shah F, Bosch FX, Muñoz N, Gissmann L, Shah KV (1992). Antibodies to HPV-16 E6 and E7 proteins as markers for HPV-16-associated invasive cervical cancer. Virology.

[41] Kim JH, Cho IH, Seo SM, Kim JS, Oh KH, Kang HS, Kim IG, Paek SH (2009). Immuno-chromatographic analysis for HPV-16 and 18 E7 proteins as a biomarker of cervical cancer caused by human papillomavirus. Bull Kor Chem Soc.

[42] Metzger C, Pittl A, Kaufmann AM, Agorastos T, Chatzistamatiou K, Böcher O (2016). A new sandwich ELISA test simultaneously detecting E7 proteins of HPV-16, 18 and 45 in cervical smears. Clin Microbiol.

[43] Gariglio P, Organista-Nava J, Alvarez-Rios E (2005). Role of HR-HPVs E6 and E7 oncoproteins in cervical carcinogenesis. J Mol Genet Med.

[44] Meschede H, Zumbach K, Braspenning J, Scheffner M, Benitez-Bribiesca L, Luande J, Gissmann L, Pawlita M (1998). Antibodies against early proteins of human papillomaviruses as diagnostic markers for invasive cervical cancer. J Clin Microbiol.

[45] Seigneuric R, Mjahed H, Gobbo J, Joly AL, Berthenet K, Shirley S, Garrido C (2011). Heat shock proteins as danger signals for cancer detection. Front Oncol.

[46] Gunaldi M, Kocoglu H, Okuturlar Y, Gedikbasi A, Karabulut M, Alis H, Hursitoglu M (2015). Heat shock protein 70 is a useful marker for predicting colorectal cancer. JBUON.

[47] Shan N, Zhou W, Zhang S, Zhang Y (2016). Identification of HSPA8 as a candidate biomarker for endometrial carcinoma by using iTRAQ-based proteomic analysis. Onco Targets Ther.

[48] Khan R, Siddiqui NN, Haq A, Rahman MA (2016). Introducing differential expression of human heat shock protein 27 in hepatocellular carcinoma: moving toward identification of cancer biomarker. Tumor Biol.

[49] Tweedle EM, Khattak I, Ang CW, Nedjadi T, Jenkins R, Park BK, Kalirai H, Dodson A, Azadeh B, Terlizzo M, Grabsch H, Mueller W, Myint S, Clark P, Wong H, Greenhalf W, Neoptolemos JP, Rooney PS, Costello E (2010). Low molecular weight heat shock protein HSP27 is a prognostic indicator in rectal cancer but not colon cancer. Gut.

[50] Kang SH, Kang KW, Kim KH, Kwon B, Kim SK, Lee HY, Kong SY, Lee ES, Jang SG, Yoo BC (2008). Upregulated HSP27 in human breast cancer cells reduces Herceptin susceptibility by increasing Her2 protein stability. BMC Cancer.

[51] Paricharttanakul NM, Saharat K, Chokchaichamnankit D, Punyarit P, Srisomsap C, Svasti J (2016). Unveiling a novel biomarker panel for diagnosis and classification of well-differentiated thyroid carcinomas. Oncol Rep.

[52] Okuno M, Adachi S, Kozawa O, Shimizu M, Yasuda I (2016). The clinical significance of phosphorylated heat shock protein 27 (HSPB1) in pancreatic cancer. Int J Mol Sci.

[53] Korneeva I, Caputo TA, Witkin SS (2002). Cell-free 27 kDa heat shock protein (HSP27) and HSP27-cytochrome C complexes in the cervix of women with ovarian or endometrial cancer. Int J Cancer.

[54] Ghayour-Mobarhan M, Saber H, Ferns GA (2012). The potential role of heat shock protein 27 in cardiovascular disease. Clin Chim Acta.

[55] Martin-Ventura JL, Duran MC, Blanco-Colio LM, Meilhac O, Leclercq A, Michel JB, Jensen ON, Hernandez-Merida S, Tunon J, Vivanco F, Egido J (2004). Identification by a differential proteomic approach of heat shock protein 27 as a potential marker of atherosclerosis. Circulation.

[56] Rus KR, Fajs L, Korva M, Avsic-Zupanc T (2016). HMGB1 is a potential biomarker for severe viral hemorrhagic fevers. PLoS Negl Trop Dis.

[57] Lee H, Song M, Shin N, Shin CH, Min BS, Kim HS, Yoo JS, Kim H (2012). Diagnostic significance of serum HMGB1 in colorectal carcinomas. PLoS One.

[58] Pilzweger C, Holdenrieder S (2015). Circulating HMGB1 and RAGE as clinical biomarkers in malignant and autoimmune diseases. Diagnostics.

