# Supplementary figures and images for: Significance of serum antibodies against HPV E7, Hsp27, Hsp20 and Hp91 in Iranian HPV-exposed women
Source: BMC Infect Dis. 2019 Feb 12;19:142. doi: 10.1186/s12879-019-3780-2 (PMC6373072; doi:10.1186/s12879-019-3780-2)

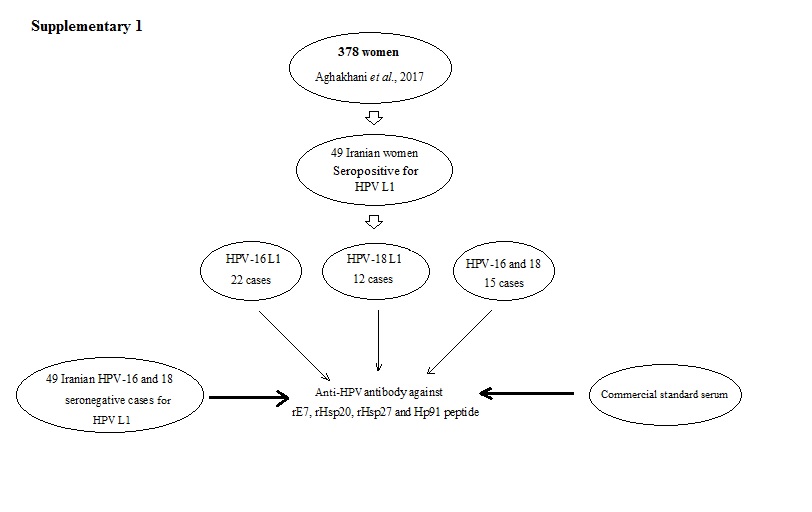

Supplement: Supplementary file 1 — Schematic representation of study population. (JPG 51 kb) [file 12879_2019_3780_MOESM1_ESM.jpg]

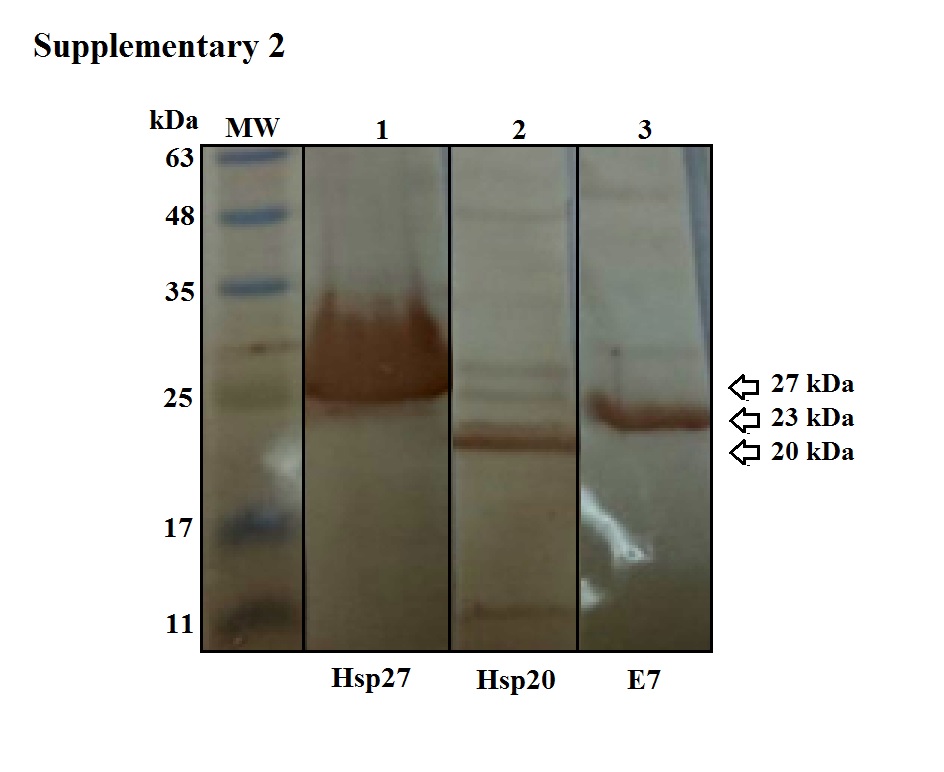

Supplement: Supplementary file 2 — Identification of the purified proteins by Western Blot analysis using anti-His antibody: lane 1: Hsp27, lane 2, Hsp20; lane 3, E7; MW is the molecular weight marker (prestained protein ladder: 10–170 kDa, Fermentase). (JPG 78 kb) [file 12879_2019_3780_MOESM2_ESM.jpg]
